# Microgravity- induced organ and system-level deconditioning: a network physiology perspective

**DOI:** 10.3389/fnetp.2026.1931834

**Published:** 2026-08-28

**Authors:** Rashmi Neginhal, Rebecca Rejo George

**Affiliations:** Department of Accredited Training Program (FCHS-ATP), Fatima College of Health Sciences, Ajman, United Arab Emirates

**Keywords:** cardiorespiratory coupling, cardiovascular, countermeasures, dynamic organ coupling, intermuscular networks, microgravity, musculoskeletal, network physiology

## Abstract

Microgravity removes the gravitational loading that anchors fluid distribution, mechanotransduction, vestibular calibration, and vascular homeostasis, exposing the human body to one of its most profound physiological challenges. Rather than producing isolated organ-specific changes, it induces a deeply interconnected, multi-system deconditioning response emerging from disrupted inter-organ communication. This narrative mini review synthesizes evidence from human spaceflight missions, ground-based analog models, and cellular studies to characterize organ- and system-level adaptations from a network physiology perspective. Skeletal muscle atrophy begins within days, with strength loss preceding structural loss due to early neural contributions. Bone mineral density falls 1%–2% per month at weight-bearing sites, with incomplete recovery after return to Earth. Cardiac remodeling, plasma volume contraction, and impaired baroreflex gain converge to produce post-flight orthostatic intolerance. Cephalad fluid redistribution elevates intracranial pressure and contributes to Spaceflight-Associated Neuro-Ocular Syndrome. Thymic involution and macrophage dysfunction impair immunity, while gut microbiome dysbiosis generates systemic inflammatory and metabolic consequences via the gut-brain axis. Endocrine shifts—including insulin resistance, cortisol dysrhythmia, and hypercalciuria—constitute an interrelated metabolic syndrome of spaceflight. These changes do not occur in isolation. Myokine-osteokine crosstalk, inflammatory amplification across the muscle-bone-gut axis, and gut-derived metabolites disrupting liver-brain metabolism represent proposed mechanisms of network-level deconditioning propagation. Critically, dynamic inter-organ coupling during spaceflight has not yet been directly measured—a fundamental research gap. Terrestrial Network Physiology studies of intermuscular and cardiorespiratory coupling networks serve as methodological precedents for future in-flight investigations. Any proposed network reconfiguration framework is explicitly hypothetical. Future research must prioritize integrated, sex-specific, network-aware countermeasure design and multi-omics biomarker development for long-duration exploration missions.

## 1 Introduction

Since the earliest human spaceflights, physicians and physiologists have recognized that sustained microgravity exposure induces profound multi-system deconditioning. What was initially framed as isolated organ-specific changes—muscle wasting, bone loss, cardiovascular deterioration—has over 6 decades of research revealed itself to be a deeply interconnected phenomenon (Bosutti et al., 2025; Hu et al., 2025; Furukawa et al., 2021). The concept of network physiology, which examines how organ systems dynamically interact and how perturbations propagate across physiological networks, provides a powerful framework for understanding why deconditioning in one organ amplifies dysfunction in others and why single-system countermeasures so frequently fall short (Goswami et al., 2026).

### 1.1 Network physiology: definition and distinction from systems biology

Network Physiology introduced by Bashan, Bartsch, Kantelhardt, Havlin, and Ivanov, quantifies dynamic, time-varying functional interactions among physiological systems (Bashan et al., 2012; Ivanov, 2021). It complements but is distinct from systems biology and molecular cross-talk. System biology commonly examines molecular and cellular interaction networks across biological scales, whereas endocrine and inflammatory cross-talk describes biochemical signaling between organs. Network Physiologymeasures the real-time, directional coordination of organ-level output signals—for example, the dynamic synchronisation between heart rate and respiratory fluctuations (cardiorespiratory coupling), the coherent co-activation of muscle groups across frequency bands (intermuscular networks), or directional information flow from brain rhythms to cardiac and locomotor systems. These interactions are captured from multichannel physiological time-series—cardiac, respiratory, electromyographic, electroencephalographic—using methods such as phase synchronisation, amplitude-amplitude cross-frequency coupling (ACFC), time-delay stability analysis, and transfer entropy (Bashan et al., 2012; Ivanov, 2021). A foundational finding is that each distinct physiological state, such as wakefulness, sleep, exercise, ageing, diseases characterized by a unique topology of inter-organ coupling, and that transitions between states involve rapid, coordinated network reorganisation on timescales of minutes. Critically, perturbations in one network node propagate through coupling links to produce emergent multi-system dysfunction disproportionate to any single-organ change—a mechanism highly relevant to microgravity-induced deconditioning.

Microgravity eliminates the omnipresent mechanical loading that on Earth constrains fluid distribution, regulates mechanotransduction in bone and muscle, maintains vestibular and proprioceptive calibration, and anchors hydrostatic gradients determining vascular tone (Mendes Zambetta et al., 2024; Shahzad et al., 2025). Its removal triggers cascading adaptations that, while appropriate for the short-term space environment, become clinically significant over months or years (Mendes Zambetta et al., 2024; Jordan et al., 2022). The International Space Station (ISS) has provided an invaluable platform for characterizing these changes, with emerging 1-year mission data illuminating longer-term deconditioning trajectories (Mozalbat et al., 2025).

Ground-based analogue models—including head-down bed rest (HDBR), dry immersion, and rodent hindlimb unloading—have enabled controlled mechanistic investigations not feasible during actual spaceflight (Marusic et al., 2021; Blaber et al., 2022; Saveko et al., 2023). Together with advances in multi-omics and computational physiology, these models have generated an increasingly integrated picture of microgravity-induced deconditioning (Magni et al., 2025).

A recent perspective article by Goswami et al., formally articulated the vision for Network Physiology in Space, establishing its theoretical foundations, analytical toolkit, and multi-scale scope—from genomic networks to organ-system dynamics—and called for multichannel in-flight data collection as the foundational next step (Goswami et al., 2026). The present mini review complements that perspective by providing the organ-system-level mechanistic evidence base that Network Physiology in Space investigations will need to contextualise and interpret. The uniqueness of this mini review lies in its integration of three key aspects that have not been collectively addressed in previous literature: (i) a systematic synthesis of what is currently known about deconditioning in each physiological system; (ii) an explicit clarification that the existing molecular, endocrine, and inflammatory cross-talk evidence does not constitute direct measurement of dynamic inter-organ coupling; and (iii) a proposal of specific terrestrial Network Physiology findings—intermuscular, cardiorespiratory, and cardiomuscular network studies—as methodological precedents and as the basis for hypotheses to be tested in future in-flight research.

This mini review synthesizes organ- and system-level adaptations to microgravity, organized by physiological domain while explicitly highlighting inter-system connections. We address the musculoskeletal, cardiovascular-autonomic, neurovestibular-central nervous, respiratory, immune, endocrine-metabolic-renal, gastrointestinal-microbiome, and reproductive systems before integrating findings under a network physiology framework.

## 2 Organ- and system-level adaptations to microgravity-induced deconditioning

### 2.1 Musculoskeletal system adaptations

#### 2.1.1 Skeletal muscle atrophy

Skeletal muscles are exquisitely sensitive to mechanical loading. Removal of gravitational loading during spaceflight or HDBR rapidly initiates a catabolic program characterized by suppression of anabolic signaling pathways, upregulation of ubiquitin-proteasome and autophagy-lysosome proteolytic cascades, and progressive myofiber loss (Bosutti et al., 2025; Hu et al., 2025).

Bosutti et al. synthesized evidence from spaceflight and bed-rest studies demonstrating rapid skeletal muscle atrophy, reduced cross-sectional area, fiber-type transitions from slow-to fast-twitch, impaired mitochondrial function, insulin resistance, and diminished strength and endurance (Bosutti et al., 2025). JAXA investigations by Furukawa et al. confirmed marked atrophy of antigravity muscles, particularly the soleus, with upregulated protein degradation pathways—explicitly paralleling age-related sarcopenia (Furukawa et al., 2021).

A systematic review by Marusic et al. characterizing neuromuscular deconditioning across 5–120 days of bed rest in 318 healthy adults found a strength-loss to atrophy ratio of approximately 4.2:1 at day 5, declining to 1.9:1 after 35 days, implicating disproportionate early neural contributions—impaired motor unit recruitment and altered neuromuscular drive—before structural atrophy becomes dominant (Marusic et al., 2021). Roberts et al. demonstrated that catabolic responses are evolutionarily conserved, mediated by myokines and osteokines, and particularly severe in tendon tissue where extracellular matrix remodeling is impaired (Roberts et al., 2025).

At the mitochondrial level, chronic disuse reduces biogenesis, impairs mitophagy, and alters fission-fusion dynamics, allowing defective mitochondria to accumulate reactive oxygen species (ROS) that amplify atrophy through proteolytic activation (Memme et al., 2021). Theilen et al. proposed combined exercise and mitochondrial transcription factor A (*TFAM*)-targeted strategies as preventive countermeasures (Theilen et al., 2017), while Graham et al. elucidated resistance training’s epigenetic adaptations counteracting muscle wasting across unloading conditions (Graham et al., 2021).

#### 2.1.2 Bone loss

Astronauts lose approximately 1%–2% of bone mineral density (BMD) per month, predominantly at weight-bearing skeletal sites including the proximal femur, femoral neck, and lumbar spine (Man et al., 2022). Man et al. confirmed increased osteoclast-mediated resorption and suppressed osteoblast function as principal drivers, with trabecular microarchitectural recovery remaining incomplete for months to years’ post-flight (Man et al., 2022).

A murine ISS study by Cahill et al. exposing female C57BL/6J mice to 37 days of spaceflight demonstrated site-specific bone loss exclusively at weight-bearing sites: femoral head bone volume fraction decreased 27%, distal femoral epiphyseal BV/TV 55%, and midshaft cortical thickness 8%, whereas lumbar vertebral bone showed no significant loss—providing strong evidence that mechanical unloading, not ionizing radiation, is the primary driver during low-Earth orbit missions (Cahill et al., 2025).

Bonanni et al. highlighted the interplay between ROS accumulation and impaired osteoblast survival, suggesting antioxidant strategies as complements to exercise countermeasures (Bonanni et al., 2023).

#### 2.1.3 Musculoskeletal countermeasures

Current ISS exercise countermeasures—principally the Advanced Resistive Exercise Device (ARED) with aerobic modalities—mitigate but do not fully prevent musculoskeletal deconditioning. Hu et al. reviewed blood flow restriction training (BFRT) as a promising adjunct achieving adaptations comparable to high-intensity resistance training with substantially reduced hardware mass (Hu et al., 2025), while Bosutti et al. highlighted neuromuscular electrical stimulation (NMES) and artificial gravity as complementary modalities with unresolved dosing and sex-specific response questions (Bosutti et al., 2025).

### 2.2 Cardiovascular and autonomic system adaptations

#### 2.2.1 Cardiac structural remodeling

Microgravity initiates a biphasic cardiovascular response. Acutely, 1–2 L of blood redistributes cephalad, transiently elevating cardiac preload; compensatory diuresis then contracts plasma volume 10%–15%, normalizing cardiac output at the cost of reduced circulating volume (Mendes Zambetta et al., 2024; Komorowski et al., 2016). Over longer durations, myocardial atrophy and reduced left ventricular mass develop. Shahzad et al. identified endothelial dysfunction, cytoskeletal reorganization, altered vascular permeability, coagulation abnormalities, and altered vasoconstrictor responses as concurrent processes increasing cardiovascular risk (Shahzad et al., 2025). Sy et al. highlighted calcium-handling dysregulation—reduced nitric oxide synthase activity, altered *RyR2* phosphorylation increasing arrhythmia susceptibility, and modified calcium-handling protein expression—with direct implications for in-flight arrhythmia risk (Sy et al., 2023).

The meta-analysis by Mendes Zambetta et al., integrating 62 studies (963 participants), confirmed increased heart rate, reduced stroke volume and cardiac output, decreased systolic blood pressure, jugular venous distension, carotid artery remodeling, and impaired heart rate variability across spaceflight and simulated microgravity conditions (Mendes Zambetta et al., 2024).

#### 2.2.2 Vascular and autonomic adaptations

Navasiolava et al. documented endothelial dysfunction, reduced flow-mediated dilation, arterial stiffening, and structural vascular remodeling reflecting loss of pulsatile shear stress signaling (Navasiolava et al., 2020). Alzaabi et al. noted that cephalad fluid shifts raise intracranial pressure (ICP) and alter cerebral venous outflow, creating conditions for long-duration neurological complications (Alzaabi et al., 2025). Takahashi et al. identified oxidative stress, driven by microgravity and space radiation, as accelerating ageing-like vascular changes at approximately 10-fold the terrestrial rate (Takahashi et al., 2017).

Post-flight orthostatic intolerance affects a substantial proportion of astronauts returning from long-duration missions. Jordan et al.’s review of 120 studies implicated plasma volume reduction, impaired baroreflex control, altered sympatho-parasympathetic balance, and reduced vascular responsiveness (Jordan et al., 2022). Blaber et al. demonstrated in 27 astronauts and 19 HDBR participants that weakened neural blood pressure-to-heart rate coupling underlies this intolerance, with females showing lower neural-mechanical causality—suggesting a need for sex-specific countermeasure protocols (Blaber et al., 2022). Goswami et al. affirmed that artificial gravity via short-radius centrifugation is the only countermeasure theoretically capable of simultaneously addressing multiple cardiovascular, musculoskeletal, and fluid-shift deconditioning targets (Goswami et al., 2026).

### 2.3 Neurovestibular and central nervous system adaptations

#### 2.3.1 Sensorimotor and vestibular reorganization

The vestibular system confronts a fundamentally altered afferent environment in microgravity: otolith organs evolved to sense the 1 g gravitational vector must recalibrate in its absence (Arjmand et al., 2025; Clément et al., 2020). Saveko et al. compared sensorimotor effects across HDBR, dry immersion, parabolic flight, and unilateral lower-limb suspension, documenting altered proprioception, balance deficits, gait instability, and vestibular disturbances across all models with differences in magnitude and time course (Saveko et al., 2023). Arjmand et al. reviewed neural changes including altered vestibular processing, spatial disorientation, impaired cognitive performance, and memory dysfunction, with ionizing radiation, hypoxia, and psychosocial confinement contributing additional neurological stressors (Arjmand et al., 2025). Clément et al., reviewing over 50 years of spaceflight research, identified long-duration Mars-class missions as posing unprecedented risks to sensorimotor control, cognition, and behavioral health (Clément et al., 2020).

#### 2.3.2 Intracranial pressure and Spaceflight-Associated Neuro-Ocular Syndrome

Cephalad fluid redistribution in microgravity elevates ICP and impairs cerebral autoregulation. Kermorgant et al. reviewed HDBR and dry immersion evidence of impaired autoregulation contributing to dizziness, cognitive slowing, and orthostatic intolerance (Kermorgant et al., 2020). Alzaabi et al. confirmed elevated ICP and altered cerebral blood flow as consistent spaceflight findings, with neuroplastic adaptations only partially compensating during flight (Alzaabi et al., 2025).

Hughes-Fulford et al. noted potentially different neuro-ocular susceptibility profiles between female and male astronauts, highlighting the importance of sex-specific data collection (Hughes-Fulford et al., 2024).

### 2.4 Respiratory system adaptations

The pulmonary system demonstrates paradoxical resilience to microgravity. Abolition of gravity-dependent ventilation-perfusion (V/Q) gradients produces more uniform V/Q matching and preserved gas exchange, confirmed across Spacelab, Skylab, and ISS experiments (Smith et al., 2024). Smith et al. noted that while macroscopic lung function is broadly preserved, cellular responses—including differential expression of cell adhesion molecules and extracellular matrix components—remain understudied and may alter epithelial host-defense mechanisms (Smith et al., 2024). Immune dysregulation discussed below substantially increases respiratory infection susceptibility, of particular concern beyond low-Earth orbit where rapid evacuation is not feasible. Miranda et al. highlighted additional pulmonary risks from lunar dust inhalation on planetary surface operations, noting the sharp crystalline structure and reactive surface chemistry of lunar regolith make it potentially more hazardous than terrestrial mineral dusts (Miranda et al., 2023).

### 2.5 Immune system adaptations

#### 2.5.1 Innate immunity

Microgravity profoundly disrupts innate immune function. Sun et al. documented macrophage cytoskeletal compromise, altered *ICAM-1* expression, and dysregulated macrophage polarization impairing inflammatory responses and intercellular communication (Sun et al., 2021). Concurrently, microgravity alters microbial behavior: Ocampo et al. demonstrated that *Enterobacter cloacae* undergoes significant proteomic adaptation under simulated low-shear microgravity, potentially shifting from commensal to pathogenic behavior in immunocompromised hosts (Ocampo et al., 2025).

#### 2.5.2 Adaptive immunity and thymic involution

Muramatsu et al. reviewed the impact of microgravity, ionizing radiation, circadian disruption, and psychosocial stress on thymic structure, documenting accelerated involution that impairs thymopoiesis and reduces naïve T-cell output—increasing susceptibility to novel pathogens and diminishing vaccine responses (Muramatsu et al., 2024). Minoretti et al. proposed multi-system biomarker panels spanning musculoskeletal, cardiovascular, immune, neurological, and gastrointestinal domains to enable personalized countermeasure selection (Minoretti et al., 2024). Oxidative stress further contributes to immune senescence by damaging lymphocyte DNA and impairing antioxidant defenses (Takahashi et al., 2017).

### 2.6 Endocrine, metabolic, and renal system adaptations

Magni et al. framed spaceflight-associated endocrine, metabolic, and renal alterations within the concept of the ‘space exposome’—the totality of microgravity, radiation, confinement, and circadian disruption exposures (Magni et al., 2025). Key findings include insulin resistance (driven by inactivity, altered adipokine secretion, and gut dysbiosis), dyslipidemia, HPA-axis dysregulation with altered cortisol rhythmicity, hypercalciuria, suppressed parathyroid hormone, and elevated bone resorption markers—constituting an interrelated metabolic syndrome of spaceflight rather than isolated perturbations. Initial cephalad fluid shifts suppress antidiuretic hormone, drive diuresis and plasma volume contraction, and create calciuric stress increasing renal calculus risk (Magni et al., 2025). Komorowski et al. highlighted that these fluid-electrolyte adaptations alter drug pharmacokinetics with implications for in-flight medical care (Komorowski et al., 2016).

Albi et al. reviewed microgravity-induced alterations in thyroid cell morphology, gene expression, cytoskeletal organization, and thyrotropin receptor sensitivity, with changed parafollicular C-cell calcitonin production having downstream consequences for calcium homeostasis and bone remodeling (Albi et al., 2017). Mozalbat et al. linked microgravity-induced metabolic changes—confirmed in the NASA Twins Study and ISS experiments—to hallmarks of biological aging including telomere shortening, genomic instability, and cellular senescence, with partial reversal upon return to Earth suggesting the spaceflight-recovery cycle as a model for anti-aging research (Mozalbat et al., 2025).

### 2.7 Gastrointestinal and gut microbiome adaptations

The gut microbiome is increasingly recognized as a central regulator of systemic physiology, influencing immune function, metabolic health, cardiovascular tone, and central nervous system function via the gut-brain axis (Ibrahim et al., 2024; Xiong et al., 2025). Ibrahim et al. reviewed microgravity-associated dysbiosis characterized by an elevated Firmicutes/Bacteroidetes ratio, increased lipopolysaccharide (LPS) promoting systemic inflammation, elevated p-cresol and secondary bile acids, and reduced abundance of cardioprotective Akkermansia muciniphila—proposed to drive endothelial dysfunction, muscle catabolism, metabolic disturbances, and cardiovascular impairment via systemic mediators (Ibrahim et al., 2024).

Xiong et al. exposed mice to simulated microgravity via 3D-clinostat and performed 16S rRNA sequencing, non-targeted serum metabolomics, and fecal microbiota transplantation (FMT) experiments (Xiong et al., 2025). Simulated microgravity produced dysbiosis, altered serum metabolite profiles, disrupted hepatic lipid, amino acid, and glucose metabolism, and disturbed brain neurotransmitter and hormone secretion. FMT recipients recapitulated hepatic and brain metabolic changes, confirming that dysbiosis per se—not other aspects of microgravity—mediates liver-brain metabolic dysfunction, with valeric acid emerging as a candidate gut-derived mediator (Xiong et al., 2025).

### 2.8 Reproductive system adaptations

The reproductive system has received comparatively limited attention in spaceflight research (Hughes-Fulford et al., 2024; Mishra and Luderer, 2019). Mishra and Luderer reviewed reproductive hazards of space travel, noting high-energy particle radiation sensitivity of ovarian follicles and spermatogonia; spaceflight and simulated microgravity disrupt spermatogenesis and suppress testicular testosterone synthesis in rodent models, while sparse female data document disrupted estrous cycling and impaired follicle development (Mishra and Luderer, 2019). Magni et al. noted that the hypothalamic-pituitary-gonadal axis is altered by the space exposome, with clinical significance for human fertility after long-duration missions remaining poorly characterized (Magni et al., 2025). Hughes-Fulford et al. highlighted the critical importance of expanding female-specific spaceflight physiology research given women’s underrepresentation in the astronaut physiological database (Hughes-Fulford et al., 2024).

## 3 A network physiology perspective

The organ-by-organ review above is useful for understanding mechanisms, but it should not obscure the key point: microgravity deconditioning is a network-wide phenomenon, not a series of separate organ failures. Network physiology examines how organ systems couple dynamically through neural, endocrine, metabolic, and mechanical signalling pathways, and how perturbations in one node propagate through the network to produce emergent multi-system dysfunction. Microgravity is perhaps the clearest natural experiment in disruption of this physiological network.

### 3.1 From molecular cross-talk to dynamic coupling: recognising the empirical gap

The cross-system interactions described above, including myokine-mediated bone effects, gut LPS-driven muscle catabolism, HPA-axis cortisol suppression of lymphocytes, gut-derived metabolites disrupting liver-brain metabolism, represent molecular, endocrine, and inflammatory cross-talk. These are important and well-supported mechanisms. However, they do not constitute empirical evidence of disrupted dynamic functional coupling in the Network Physiology sense (Bashan et al., 2012; Ivanov, 2021). In Network Physiology, inter-organ coupling is characterised by time-varying, directional interactions between the output signals of physiological systems, measured by methods such as ACFC, phase synchronisation, time-delay stability, and transfer entropy. A fundamental and underappreciated research gap is the complete absence of simultaneous, multichannel dynamic coupling investigations during actual spaceflight or ground-based analogues. The statements in this review about cross-system network disruption should therefore be understood as biologically motivated hypotheses—grounded in molecular, structural, and autonomic evidence—rather than as empirically demonstrated changes in dynamic inter-organ coupling. The proposition that microgravity degrades coupling strength, alters network topology, or reduces network flexibility remains to be directly tested.

### 3.2 Terrestrial Network Physiology studies as methodological precedents

Terrestrial Network Physiology provides both the analytical methods and specific empirical precedents for future spaceflight investigations. The foundational study by Bashan et al. demonstrated that the human organism is characterised by distinct functional interaction networks among cerebral, cardiac, respiratory, ocular, and locomotor systems that undergo rapid state-dependent reorganisation on timescales of minutes (Bashan et al., 2012). Ivanov subsequently expanded this framework toward building the Human Physiolome—a comprehensive atlas of dynamic network interactions across physiological states and conditions (Ivanov, 2021).

In the context most directly relevant to spaceflight deconditioning, Garcia-Retortillo et al. demonstrated using ACFC-derived intermuscular network analysis during a squat-to-exhaustion exercise protocol—that ageing is associated with a significant breakdown in networks of intermuscular coordination: reduced coupling strength across frequency bands between agonist-antagonist and bilateral muscle pairs, predictive of functional decline beyond what single-muscle assessments detect (Garcia-Retortillo et al., 2025a). This ageing-related intermuscular network breakdown closely parallels the sarcopenic phenotype that Furukawa et al. explicitly linked to spaceflight (Furukawa et al., 2021), making intermuscular coupling network degradation a plausible and directly testable consequence of microgravity-induced muscle unloading. A follow-up case report further demonstrated that a 12-week structured exercise programme produced a ∼400% increase in average intermuscular network link strength, indicating that Network Physiology coupling metrics are sensitive to intervention-induced recovery in ways that conventional single-muscle assessments may not be (Garcia-Retortillo et al., 2025b).

Similarly, terrestrial studies of cardiorespiratory coupling—dynamic synchronisation between cardiac and respiratory oscillations—and cardiomuscular networks—coupling between cardiac output dynamics and skeletal muscle activation during exercise and fatigue—have identified state-specific coupling signatures that degrade with ageing and fatigue, and reorganise with training (Bashan et al., 2012; Ivanov, 2021). The weakening of neural blood pressure-to-heart rate coupling documented by Blaber et al. in astronauts after spaceflight (Blaber et al., 2022) represents, in principle, exactly the directional cardiovascular-autonomic coupling disruption that Network Physiology methods are designed to quantify, though it was not analyzed within that framework. Reanalysis of existing spaceflight cardiovascular time-series using transfer entropy or time-delay stability methods could provide an initial retrospective estimate of in-flight coupling changes without requiring new data collection. Ground-based analogues such as HDBR and dry immersion are the ideal controlled first step for applying these methods to a deconditioning model (Saveko et al., 2023).

### 3.3 A proposed hypothetical framework and the four network coupling chains

On the basis of the molecular, structural and autonomic evidence reviewed above and terrestrial Network Physiology precedents, we propose a hypothetical framework for how microgravity may influence inter-organ coordination. This framework is explicitly presented as a set of testable propositions, not as established findings. Mitochondrial dysfunction is positioned in this framework as a mechanism contributing to dysfunction at several individual network nodes—not as a central hub of the inter-organ network itself, for which no direct empirical evidence exists. The four proposed molecular coupling chains are presented below as mechanistic hypotheses to be tested by future multimodal, multichannel in-flight investigations using Network Physiology analytical methods.

#### 3.3.1 Muscle-bone-gut axis

Muscle atrophy reduces myokine secretion (irisin, myostatin), diminishing osteoblastic stimulation and exacerbating bone loss (Roberts et al., 2025). Simultaneously, gut dysbiosis with elevated LPS drives systemic inflammation accelerating muscle catabolism and osteoclast activation (Ibrahim et al., 2024), creating a self-amplifying spiral compounding deconditioning across three organ systems.

#### 3.3.2 Cardiovascular-autonomic-vestibular triad

Cardiovascular deconditioning reduces baroreceptor loading, impairing baroreflex gain and autonomic cardiovascular control (Jordan et al., 2022; Blaber et al., 2022). Cephalad fluid redistribution simultaneously elevates ICP and alters otolith afferent signals, disrupting vestibular calibration and postural motor control and proprioceptive feedback to the autonomic cardiovascular system (Saveko et al., 2023; Kermorgant et al., 2020). The resulting destabilized network leaves astronauts vulnerable to falls and syncope on return to gravity.

#### 3.3.3 Immune-endocrine-mitochondrial network

Thymic involution reduces adaptive immune surveillance (Muramatsu et al., 2024), HPA-axis dysregulation chronically suppresses lymphocyte function (Magni et al., 2025), and mitochondrial ROS generation damages immune cell DNA and amplifies inflammatory signaling (Memme et al., 2021). Combined with microbial adaptation to the space environment (Minoretti et al., 2024), these pathways produce an immune senescence state elevating infection risk in a closed environment with no rapid access to terrestrial care.

#### 3.3.4 Gut-brain-liver metabolic network

Dysbiosis-derived altered short-chain fatty acid and secondary metabolite profiles disrupt hepatic lipid, amino acid, and glucose metabolism while influencing neurotransmitter synthesis and hormone secretion via the gut-brain axis (Xiong et al., 2025). These interconnected shifts—confirmed as causally linked by FMT experiments—manifest as insulin resistance, dyslipidemia, and cognitive changes rather than independent organ dysfunctions.

**FIGURE 1:**
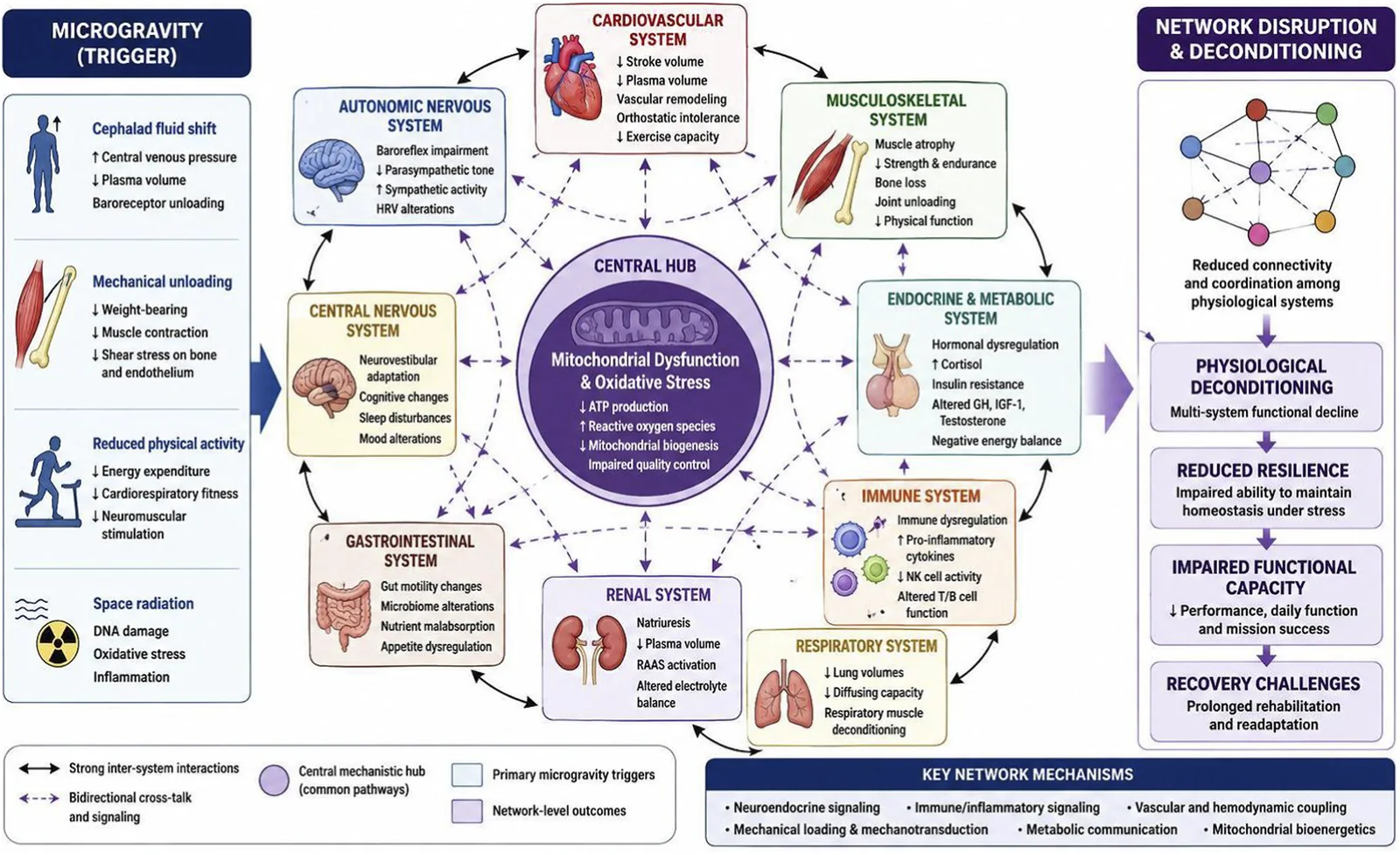
Network physiology framework of microgravity-induced deconditioning. Microgravity triggers multiple primary adaptations that initiate widespread changes across interconnected physiological systems. Mitochondrial dysfunction and oxidative stress act as central hubs, propagating disturbances through bidirectional interactions, leading to network disruption and progressive physiological deconditioning.

A network physiology lens reveals that single-system countermeasures are inherently limited. Exhaustive in-flight exercise protocols, while preserving muscle mass and cardiovascular fitness to a degree, fail to prevent bone loss at all sites, neurovestibular challenges, immune compromise, or gut dysbiosis (Ahmed et al., 2024). Future countermeasure design must account for cross-system coupling, targeting multiple network nodes simultaneously. Figure 1 presents this proposed model for future testing (Figure 1).

## 4 Discussion

This review has organized microgravity-induced deconditioning organ by organ while emphasizing that the underlying biology is a network-level phenomenon. Several themes emerge.

First, countermeasure adequacy. Ahmed et al.’s systematic review of 16 standalone passive countermeasure trials found that LBNP improved orthostatic tolerance, whole-body vibration enhanced balance, and intermittent centrifugation partially preserved cardiovascular function, but no passive countermeasure alone prevented the majority of deconditioning outcomes (Ahmed et al., 2024) BFRT (Hu et al., 2025) and artificial gravity (Goswami et al., 2026) represent the most promising next-generation modalities, with microbiome modulation (Ibrahim et al., 2024; Xiong et al., 2025) an emerging complementary strategy.

Second, sex-specific physiology remains inadequately characterized. The majority of spaceflight data derive from male astronauts, yet evidence for meaningful sex differences in orthostatic intolerance, bone loss susceptibility, neuro-ocular syndrome, and reproductive effects is accumulating (Blaber et al., 2022; Hughes-Fulford et al., 2024; Ahmed et al., 2024). Mission protocols and reference ranges require urgent sex-stratified revision.

Third, multi-omics integration is required to move beyond descriptive cataloging toward mechanistic network models. Magni et al. called for integrated genomic, proteomic, metabolomic, and microbiome datasets from longitudinal astronaut studies analyzed with machine learning tools to identify cross-system coupling signatures (Magni et al., 2025). Minoretti et al.’s multi-system biomarker panel concept aligns with this network approach by treating deconditioning risk as a whole-network property (Minoretti et al., 2024).

Fourth, the translational potential for terrestrial medicine is substantial. Parallels between spaceflight deconditioning and aging, immobilization syndrome, and critical illness are numerous and mechanistically coherent (Furukawa et al., 2021; Mozalbat et al., 2025). Insights from space physiology could advance rehabilitation medicine, gerontology, and critical care, justifying sustained public investment in this research program independent of immediate crewed deep-space mission planning.

Finally, the transition from 6-month ISS missions to multi-year Mars-class missions will expose astronauts to deconditioning trajectories that exceed current experience, with no option for early return and limited countermeasure access. Urgently required are: integrated network-aware countermeasure designs, sex-specific protocols, multi-omics longitudinal datasets, validated in-flight biomarker panels, and physiological modeling frameworks capable of predicting individual deconditioning trajectories and therapeutic tipping points (Table 1) (Goswami et al., 2026; Magni et al., 2025).

**TABLE 1:** Critical research gaps and future research priorities in microgravity-induced deconditioning.

Research domain	Current knowledge gaps	Recommended approaches/priorities
Sex differences	Limited female astronaut data; mechanisms of greater orthostatic intolerance and bone loss in women unclear	Recruit more female participants; longitudinal studies; mechanistic investigations; sex-specific countermeasure development
Individual variability	Genetic and molecular determinants of deconditioning severity unknown; predictors lacking	Genomic/proteomic profiling; machine learning models; pre-flight biomarker screening; personalized countermeasures
Neural mechanisms	Early strength loss vs. atrophy relationship incompletely understood; neural plasticity mechanisms unclear	Longitudinal neuroimaging; electrophysiology; detailed neuromuscular testing; neural rehabilitation approaches
Cross-system integration	Specific feedback loops and coordinated signalling between organ systems incompletely mapped	Integrated multi-omics; computational modelling; systems biology approaches; longitudinal integrated phenotyping
Gut microbiota role	Causal links between dysbiosis and organ dysfunction; optimal microbiota-targeted interventions unknown	Faecal microbiota transplant validation; probiotic/prebiotic trials; metabolite supplementation testing; mechanism studies
Countermeasure optimization	Suboptimal efficacy; lack of standardized outcomes; equipment/infrastructure limitations unresolved	Higher-quality RCTs; standardized physiological endpoints; innovative low-mass technologies; dose-response studies
Recovery mechanisms	Reasons for incomplete bone and some cardiovascular recovery post-flight unclear; time course variable	Post-flight longitudinal monitoring; molecular recovery pathway investigation; rehabilitation optimization
Long-duration effects	Limited data on missions >6–12 months; cumulative exposure effects; Mars mission duration risks unknown	Extended ISS missions with comprehensive monitoring; computational models; long-duration analog studies

## 5 Conclusion

Microgravity-induced deconditioning is not just a series of changes in specific organs. It disrupts physiological networks in a coordinated way. Mechanical unloading, fluid shifting toward the head, changes in autonomic function, immune system issues, metabolic changes, and alterations in gut bacteria all interact across different biological levels, leading to widespread dysfunction in multiple systems. Looking at this from a network physiology perspective offers a better way to understand spaceflight deconditioning and to create integrated countermeasures. Future research should focus on long-term, sex-specific studies that examine various biological layers and use computational models to identify individual vulnerability patterns. This can help in developing personalized strategies for countermeasures during long space missions.
